# Molecular Characterization of the *Burkholderia cenocepacia* *dcw* Operon and FtsZ Interactors as New Targets for Novel Antimicrobial Design

**DOI:** 10.3390/antibiotics9120841

**Published:** 2020-11-24

**Authors:** Gabriele Trespidi, Viola Camilla Scoffone, Giulia Barbieri, Giovanna Riccardi, Edda De Rossi, Silvia Buroni

**Affiliations:** Department of Biology and Biotechnology “Lazzaro Spallanzani”, University of Pavia, 27100 Pavia, Italy; gabriele.trespidi01@universitadipavia.it (G.T.); viola.scoffone@unipv.it (V.C.S.); giulia.barbieri@unipv.it (G.B.); giovanna.riccardi@unipv.it (G.R.)

**Keywords:** cell division, FtsZ, *Burkholderia cenocepacia*, drug resistance, new drug targets

## Abstract

The worldwide spread of antimicrobial resistance highlights the need of new druggable cellular targets. The increasing knowledge of bacterial cell division suggested the potentiality of this pathway as a pool of alternative drug targets, mainly based on the essentiality of these proteins, as well as on the divergence from their eukaryotic counterparts. People suffering from cystic fibrosis are particularly challenged by the lack of antibiotic alternatives. Among the opportunistic pathogens that colonize the lungs of these patients, *Burkholderia cenocepacia* is a well-known multi-drug resistant bacterium, particularly difficult to treat. Here we describe the organization of its division cell wall (*dcw*) cluster: we found that 15 genes of the *dcw* operon can be transcribed as a polycistronic mRNA from *mraZ* to *ftsZ* and that its transcription is under the control of a strong promoter regulated by MraZ. *B. cenocepacia* J2315 FtsZ was also shown to interact with the other components of the divisome machinery, with a few differences respect to other bacteria, such as the direct interaction with FtsQ. Using an in vitro sedimentation assay, we validated the role of SulA as FtsZ inhibitor, and the roles of FtsA and ZipA as tethers of FtsZ polymers. Together our results pave the way for future antimicrobial design based on the divisome as pool of antibiotic cellular targets.

## 1. Introduction

Bacterial division is a very complex and spatiotemporally regulated process in which several proteins cooperate for the formation of the divisome. The process integrates DNA replication, chromosome segregation and septum formation making its full characterization very challenging [1,2]. Nevertheless, within the past 30 years many components of the divisome have been discovered and functionally characterized, in particular in the model organisms *Caulobacter crescentus* [3], *Escherichia coli* [4] and *Bacillus subtilis* [5]. In *E. coli*, division machinery formation is composed of two temporally distinct phases: the early and the late. The assembly of the essential part of the divisome is primed by the polymerization of the tubulin-like protein FtsZ at the mid cell, stabilized and anchored to the inner membrane by the interactions with FtsA and ZipA [6]. The proteins ZapA, ZapC and ZapD cross-link FtsZ polymers increasing their stability [7,8,9]. The localization of the protein complex FtsEX (the main regulator of the peptidoglycan hydrolysis at the septum for the modeling of the new cell poles) to the forming Z ring, via FtsE-FtsZ interaction, concludes the early phase [10]. Then, the late phase occurs, after a physiological programmed delay, recruiting concurrently: FtsK, a DNA translocase and a linker of the downstream proteins to the ring [11,12]; FtsQ, FtsL, FtsB, known to form a complex acting as a protein scaffold but also having regulatory activity [13]; FtsW, recently emerged as a peptidoglycan glycosyltransferase [14]; FtsI, the ring peptidoglycan transpeptidase [15]; and FtsN, triggering the activation of the machinery and starting the septation [16].

The key component of this structure is definitely FtsZ, the prokaryotic homolog of the tubulin, that shares with its eukaryotic counterpart the GTPase activity and the related ability to form dynamic polymers through head-to-tail interactions, even though the assembly occurs with an opposite polarity [17]. During the divisome assembly, FtsZ directly interacts with most of the proteins involved becoming, after the polymerization, their molecular scaffold [18]. Moreover, the FtsZ interactome includes negative modulators that prevent the Z-ring formation in wrong places, such as: MinCD, that inhibits its formation at the cell poles [19]; SlmA, that prevents its assembly over the nucleoid [20]; SulA, that blocks the polymerization of FtsZ in case of SOS response induction [21]. The role of the FtsZ polymers is not limited to the structural function, indeed they are thought to generate the force that lead to mid cell constriction [22] and guide the synthesis of septal wall through a treadmilling behavior [23,24].

In *E. coli*, the gene *ftsZ*, together with *ftsL*, *I*, *W*, *Q*, *A*, is localized within the division cell wall (*dcw*) operon, a gene cluster involved in bacterial division and cell wall synthesis [25]. This cluster is extremely conserved across bacteria, even if gene number and order change following the cell shape [26]. For this reason, the above mentioned genes are thought to be the only ones absolutely required for a successful division, since all the others, encoded outside the *dcw* region, are demonstrated to be not essential in certain conditions [4]. The importance of this cluster lies also in its complex transcriptional control, probably contributing to the spatiotemporal coordination of the division. The complexity is given by many promoters and regulatory factors within the *dcw* sequence, required to modulate the expression of different transcriptional units based on growth phase and condition [25,27]. This regulation is not fully characterized yet, but in general it follows different pattern in different organisms [28,29,30,31,32].

In several bacteria the *dcw* operon includes, often at the 5’ of the cluster, the gene encoding the transcriptional regulator MraZ, classified within the AbrB-like superfamily of DNA-binding proteins [33,34]. Although its DNA-binding ability had already been hypothesized, only recently Eraso et al. [35] demonstrated its role in the transcriptional control of the *dcw* operon in *E. coli*: by binding a repeated sequence upstream of the first gene of the operon, MraZ represses its transcription starting from the promoter P_mra_ (*mraZ1p*). This regulation represses the expression of the first 11 genes, including its own, from *mraZ* to *murC* [35]. Furthermore, MraZ was demonstrated to affect about 23% of *E. coli* gene expression during the early logarithmic phase [35]. Despite its almost ubiquitous presence in prokaryotes, MraZ has a different role in phylogenetically distant organisms, since in mycoplasmas it was described as a transcriptional activator [36].

This increasing knowledge of the division mechanisms has risen the awareness on the potentiality of this pathway as a pool of new drug targets, given the essentiality of most proteins involved. Indeed, blocking a single component of this machinery often leads to the impairment of the entire process. In a worldwide context of antimicrobial resistance spreading, the research of novel potential cellular targets is necessary. The decreasing of the antibiotic efficacy represents a threat principally for patients suffering from recurrent infections, as people with cystic fibrosis (CF). Indeed, respiratory failure caused by chronic lung infections represents the leading cause of death for these patients [37]. Many opportunistic pathogens colonize the lungs of these people but *Burkholderia cenocepacia*, a member of the *Burkholderia cepacia* complex [38], is notorious as one of the deadliest. Upon infection, it is associated in most cases to a fast decline of pulmonary function and, in up to 20% of the infected patients, it causes a fulminant necrotizing pneumonia [39]. Moreover, it can spread from patient-to-patient, as demonstrated from the devastating outbreaks in Europe, Canada, and USA [40]. The presence of several virulence factors in its uncommonly large genome [41], and the acquisition of pathogenicity islands [42] explain the high levels of virulence of the infections. Besides, the *B. cenocepacia* natural resistance to almost every available antibiotic makes its eradication extremely challenging [43,44,45]. In recent years, we found a newly synthesized benzothiadiazole derivative, C109, showing bactericidal activity at low doses against *B. cenocepacia* [46]. This compound resulted to be a potent FtsZ GTPase inhibitor, able to impair the division of a broad-spectrum of Gram-positive and -negative pathogens, given the high conservation of the molecular target across bacteria [47]. Further in-depth characterizations [48] and the development of a PEGylated nanoparticles formulation for aerosol administration [49] validated the C109 as a promising novel antibacterial compound. Moreover, the reported activity of FtsZ inhibitors against *Staphylococcus aureus* and CF Gram-negative pathogens corroborates the potential of this protein as molecular target for new antimicrobials [50]. However, also FtsZ interactors, especially the proteins encoded in the *dcw* cluster, are considered excellent candidates, since the inhibition of their activities, besides the perturbation of their interactions with FtsZ, can critically impair bacterial division, leading to bacterial death [50].

In this study, we aimed at performing the transcriptional analysis of the *dcw* operon and the characterization of its regulator MraZ in *B. cenocepacia*, using molecular techniques. We also wanted to identify the FtsZ interactors using a bacterial adenylate cyclase two-hybrid system, as well as co-sedimentation assays. The achieved results provide new insights into *B. cenocepacia* division mechanisms, focusing the attention on the essential divisome proteins transcription and on their molecular interactions, giving an overview of the key elements of this pathway.

## 2. Results

### 2.1. Characterization of the dcw Operon in B. cenocepacia J2315

#### 2.1.1. The *dcw* Cluster of *B. cenocepacia* J2315 Has the Conserved Gene Organization Shared by Rod-Shaped Bacteria

**Figure 1:**
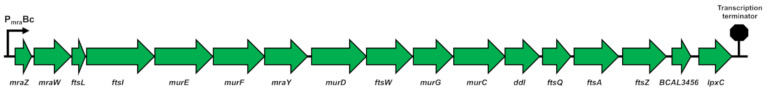
Representation of the 17 genes, and their transcriptional orientation, composing the *dcw* cluster on chromosome 1 of *B. cenocepacia* J2315. The main promoter P_mra_Bc is represented by the black arrow at the 5’ of the operon, whereas the only transcription terminator is localized at the 3’, downstream of *lpxC*.

To determine the localization and the gene organization of the *dcw* operon in *Burkholderia cenocepacia* J2315, the *Burkholderia* genome database (https://www.burkholderia.com) [51] was used. The cluster encompasses a DNA region of 19.158 bp (3803412–3784254) and is composed of 17 genes (Figure 1), localized on the negative strand with the same transcriptional polarity. It is located on chromosome 1 [41] and, despite the remarkable gene redundancy in the *B. cenocepacia* huge genome, it is present in a single copy. Regarding gene organization, it shows the same arrangement seen in *Escherichia coli*, containing the same closely packed genes from *mraZ* to *ftsZ*, with the only remarkable difference found in the insertion of the gene BCAL3456, a thioredoxin reductase, between *ftsZ* and *lpxC* (*envA*) (Figure 1).

#### 2.1.2. The dcw Operon is Transcribed as a Polycistronic mRNA from mraZ to ftsZ

In order to determine whether the *dcw* operon is subdivided into transcriptional units, its entire DNA sequence was analyzed using the bacterial rho-independent transcription terminator prediction software TransTermHP (http://transterm.ccb.jhu.edu/query.php) [52]. A transcription terminator site (3784225–3784210) was found downstream of the gene *lpxC*, but no other putative terminators were found within the operon sequence. For this reason, it has been speculated that the cluster could be transcribed as a single polycistronic mRNA. To confirm this theory, a RT-PCR approach was used. Primers were designed to test the co-transcription of gene pairs, analyzing the whole sequence from *mraZ* to *lpxC*. The RNA was extracted from cells during mid-log phase in LB medium, and subsequently retrotranscribed using the reverse primers listed in Table S1, each one designed to anneal at the 5’-end of a different *dcw* operon gene. The resulting cDNAs were used as templates in PCRs performed with the same reverse primers used for retrotranscription coupled with forward primers complementary to the 3’-end of the upstream gene. The primer pairs and the corresponding fragment lengths are reported in Table S1. The amplicons of all the gene pairs from *mraZ* to *ftsZ* were obtained, confirming that each gene is transcribed together with the flanking neighbor genes, demonstrating that in *B. cenocepacia* J2315 they can be transcribed as a single polycistronic mRNA of about 17 kb. Conversely, no fragments were amplified when cDNAs of the gene pairs *ftsZ*-*BCAL3456* and *BCAL3456-lpxC* were used as PCR templates, suggesting that the two genes are transcribed as single mRNAs. However, as mentioned above, no transcription terminators have been found downstream of the genes *ftsZ* and *BCAL3456* using the TransTermHP software, probably because a different mechanism, such as rho-dependent transcription terminators, is involved in blocking the transcription.

**Figure 2:**
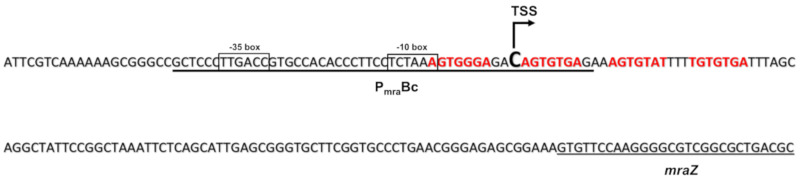
Organization of the P_mra_Bc promoter sequence. The promoter sequence (P_mra_Bc) is underlined in bold, with the −10 and −35 boxes, the cytosine representing the transcription start site (TSS) of the *dcw* operon, and the repeats of the putative MraZ binding site highlighted in red.

Subsequently, the presence of a promoter able to activate the transcription of the first 15 genes of the operon, was investigated. In *E. coli*, the transcription of the first 9 genes of the *dcw* operon starts 38 bp upstream of the *mraZ* gene and is under the direct control of the P_mra_ promoter [53]. A bioinformatic analysis of the non-coding region upstream of the *B. cenocepacia mraZ* gene (3803413–3803798) was carried out using the BDGP Neural Network Promoter Prediction (http://www.fruitfly.org/seq_tools/promoter.html), employing the default parameters for predictions of promoters in prokaryotes. The search resulted in the identification of a 50 bp putative promoter sequence starting 141 bp upstream of the first translated codon of *mraZ* (Figure 2).

**Figure 3:**
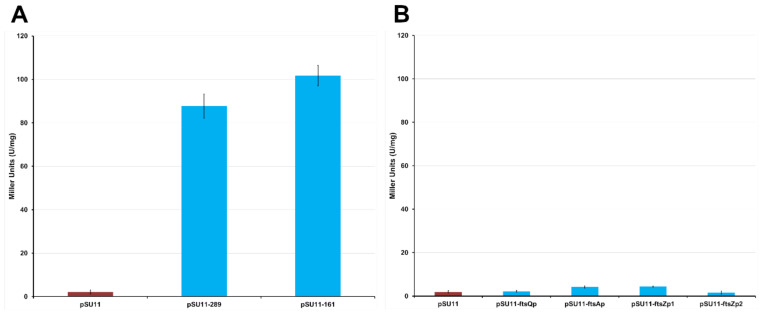
Comparison of the promoter activities of the tested fragments expressed in Miller units. (**A**) Both fragments, 289 bp (pSU11-289) and 161 bp (pSU11-161), amplified from the intergenic region upstream of *mraZ*, have a strong promoter activity, containing the promoter of the first 15 genes of the *dcw* operon P_mra_Bc. (**B**) The four fragments covering the *ddl*-*ftsA* region of the *dcw* cluster show a promoter activity comparable to the negative control (empty pSU11 vector, red bars), demonstrating the absence of additional promoters. Results are expressed as mean of 3 experiments and the error bars represent the standard deviation.

In order to experimentally confirm its promoter activity, two overlapping fragments of 289 bp and 161 bp, respectively, were amplified using the primers dcwPROMOfor1_2 and dcwPROMOrev2, and dcwPROMOfor2_2 and dcwPROMOrev2 (Table S2). These were cloned into the pSU11 vector, in which the MCS is upstream of a promoterless *lacZ* gene, obtaining pSU11-161 and pSU11-289 (Table S3). The two plasmids were transferred from *E. coli* to *B. cenocepacia* K56-2 by conjugation and the expression of LacZ was assessed by β-galactosidase activity assay, culturing the cells in LB medium and harvesting them in early stationary phase. The tested fragments had comparable activities (empty pSU11: 2.2 ± 0.92; pSU11-161: 101.8 ± 4.7 MU; pSU11-289: 87.7 ± 5.6 MU) (Figure 3A), demonstrating that the putative sequence has indeed promoter activity and that the 161 bp fragment contains the minimal transcription promoter P_mra_Bc (Figure 2) of the *dcw* operon in *B. cenocepacia*.

As the expression of *ftsZ* is known to be strictly regulated in many microorganisms [29,31,54], the presence of additional promoters in the *ddl-ftsA* region of *B. cenocepacia* J2315 was investigated. To this purpose BDGP Neural Network Promoter Prediction software was used, as previously described. From this analysis, no putative promoters were predicted, but nevertheless the sequence was experimentally tested using *lacZ* fusions. Thus, four sequential fragments were amplified: ftsQp, using murCPROMOfor1 and ddlPROMOrev1; ftsAp, using ftsQPROMOfor1 and ftsQPROMOrev1; ftsZp1, using ftsAPROMOfor1 and ftsAPROMOrev1; ftsZp2, using ftsAPROMOfor2 and ftsAPROMOrev2. In this way, the whole *ddl-ftsA* region was tested (Table S2, Supplementary Materials Figure S1). These fragments were cloned upstream of the promoterless *lacZ* gene into the pSU11 vector, creating the pSU11-ftsQp, pSU11-ftsAp, pSU11-ftsZp1, pSU11-ftsZp2 (Table S3). The promoter activity of the fragments was tested in *B. cenocepacia* K56-2 by β-galactosidase activity assay. For this experiment, the cultures were harvested when entering the stationary phase, having an OD_600_ around 2. Indeed, in *E. coli* several promoters of this region increase their activity during the stationary phase, once the growth rate decreases [54]. Despite this, all the tested sequences showed no activity (empty pSU11: 1.93 ± 0.55 MU; pSU11-ftsQp: 2.20 ± 0.38 MU; pSU11-ftsAp: 4.22 ± 0.49 MU; pSU11-ftsZp1: 4.38 ± 0.21 MU; pSU11-ftsZp2: 1.63 ± 0.71 MU) (Figure 3B), confirming that under standard laboratory culture conditions transcription starts only from the P_mra_Bc, with no intermediate promoters able to further regulate *ftsZ* expression.

Finally, the transcription start site was identified as described in Materials and Methods as a cytosine located 100 bp upstream of the first translated codon of *mraZ*, at the 3’-end of the P_mra_Bc promoter sequence (Figure 2).

#### 2.1.3. The Protein Mraz Is the Transcriptional Regulator of the dcw Operon

**Figure 4:**
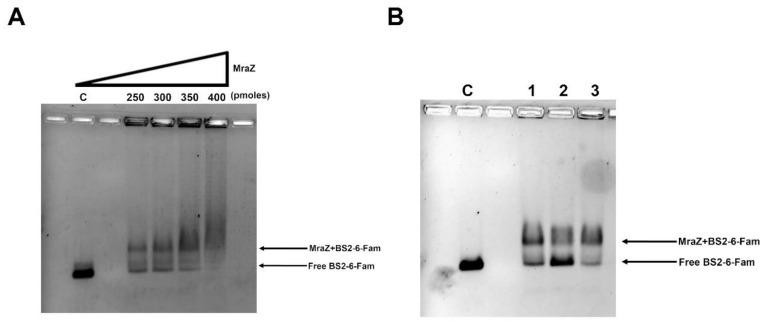
Electrophoretic mobility shift assay of the MraZ protein. (**A**) The gel represents the band shift using a constant BS2-6-Fam DNA amount (C, 0.34 pmoles) and increasing quantities of MraZ. (**B**) Demonstration of the specificity of the binding of MraZ to the fragment BS2, using a fixed amount of protein (300 pmoles) and BS2-6-Fam (0.34 pmoles) (lane 1), a 20× excess of non-labeled BS2 fragment which competes for the binding of MraZ (lane 2), or a 20× concentration of non-specific, non-labeled competitor (a 250 bp *cepI* fragment) (lane 3).

In order to identify the DNA binding site of *B. cenocepacia* MraZ, an electrophoretic mobility shift assay (EMSA) was performed. First, MraZ was expressed and purified as described in Materials and Methods. Then, two fragments were amplified using the 6-carboxyfluorescein (6-Fam) labeled primers. The two probes overlapped for 137 bp and encompassed the whole intergenic DNA region upstream of the gene *mraZ*. In particular, fragment BS1-6-Fam, containing the 5’ half of the region from −386 bp to −136 bp upstream of *mraZ* gene, was obtained using the primers mraZBS1for and mraZBS1rev (Table S2); instead, fragment BS2-6-Fam, containing the 3’ half of the region from −250 bp to −1 bp upstream of gene *mraZ*, was amplified with mraZBS2for and mraZBS2rev (Table S2). Both probes were 250 bp long and were tested in the presence of different MraZ concentrations in the assay, demonstrating that the protein is able to bind BS2-6-Fam (Figure 4A and Figure S2), but not BS1-6-Fam. The specificity of the binding was confirmed by adding a 20× excess of non-labeled BS2 fragment or a 20× excess of a non-specific 250 bp competitor in the binding reaction (Figure 4B and Figure S2). Subsequently, the BS2 fragment sequence was analyzed to find a putative MraZ DNA binding site, searching for a repeated GTG motif, known to be conserved in the binding sites of evolutionary distant bacteria, such as *B. subtilis*, *E. coli* and mycoplasmas [36]. The sequence was identified as series of four repeats of seven nucleotides separated by a 3-nucleotide spacer region (Figure 2). In particular, it is located downstream of the putative P_mra_Bc −10 box, as in *E. coli* [35], and it overlaps with the *dcw* operon transcription start site (Figure 2).

### 2.2. Study of the FtsZ Interactors in B. cenocepacia In Vivo and In Vitro

#### 2.2.1. BACTH Analysis of *B. cenocepacia* Cell Division Proteins

To explore the divisome of *B. cenocepacia*, proteins homologous to the well characterized *E. coli* cell division machinery were selected. First, the designed proteins (FtsA, SulA, ZipA, ZapA, FtsE, FtsQ, FtsI, FtsN) were tested for pairwise interactions with FtsZ using the Bacterial Adenilate Cyclase Two Hybrid (BACTH) assay. DNA fragments encoding these proteins were systematically cloned into the four vectors of the BACTH system (pUT18, pUT18C, pKT25 and pKNT25) to obtain recombinant plasmids expressing the proteins of interest fused at the C- or N-terminus of either the T18 or the T25 fragment of the *Bordetella pertussis* adenylate cyclase. To test the putative interactions between FtsZ and the other proteins, an *E. coli cya* deficient strain (BTH101) was used and co-transformed with pairs of recombinant vectors expressing the T18 and T25 protein hybrids. The functional complementation efficiencies between the hybrids were evaluated using β-galactosidase activity (in solid and in liquid media). Only the pairs of vectors that showed a significant β-galactosidase activity on solid media underwent further investigations (quantification of the β-galactosidase in liquid medium and in vitro protein-protein interaction studies).

**Figure 5:**
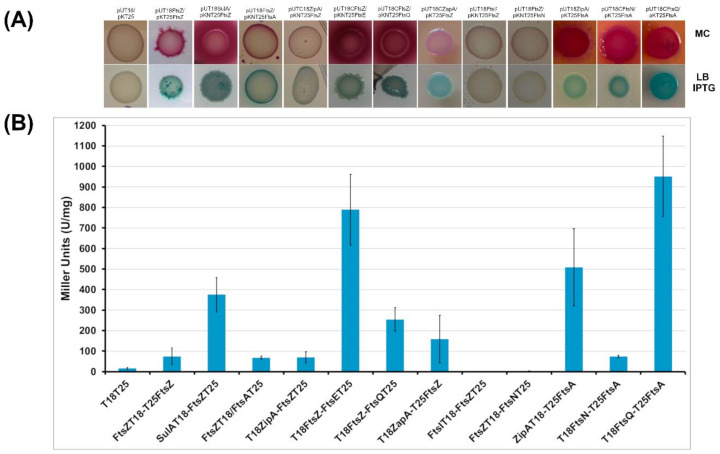
BACTH analysis of interactions between Fts proteins. (**A**) *Escherichia coli* strain BTH101 was co-transformed with two-hybrid vector plasmids (Table S3) expressing different hybrid proteins, as indicated. Transformants were spotted onto nutrient agar plates containing IPTG (LB IPTG) and X-Gal, or on MacConkey agar plates (MC) and incubated at 30 °C for 40 h. Blue coloration on LB IPTG or pink coloration on MC indicates a positive interaction. (**B**) The efficiencies of functional complementation between the indicated hybrid proteins were quantified by measuring β-galactosidase (expressed in Miller Units) activities in suspensions of *E. coli* BTH101 cells harboring the corresponding plasmids, as described in Materials and Methods. Each bar represents the mean value from results of at least three independent cultures. The *E. coli* BTH101 harboring the empty vectors was used as control.

The overexpression of the selected proteins did not affect the growth of the *E. coli* host cells (data not shown). As negative control, the *E. coli* BTH101 strain transformed with the two empty vectors pUT18 and pKT25 was used. For each protein, the four different constructs (Table S3) were used to test interaction with FtsZ by qualitative evaluation of β-galactosidase activity on solid medium. Levels of β-galactosidase activity at least four-fold higher than that measured for BTH101(pKT25/pUT18) cells were considered indicative of an interaction. As shown in Figure 5, the BTH101 strain co-transformed with the vectors both expressing FtsZ has a β-galactosidase activity five-fold higher than the control strain. This result indicates that FtsZ monomers interact each other with a head to tail interaction, as previously reported in *E. coli* [55]. Indeed, this kind of interaction is necessary for the GTPase activity and protein polymerization of FtsZ.

Among proteins involved in cell division control, in *E. coli* and in *Pseudomonas aeruginosa* SulA has been described to block FtsZ activity in case of DNA damage [56]. The putative SulA of *B. cenocepacia* is codified by the gene pBCA006. The BACTH assay showed that the putative SulA interacts using its N-terminal part with FtsZ (Figure 5A,B), with a β-galactosidase activity twenty-five-fold higher than the control. These results confirmed the involvement of the protein in the mechanism of cell division.

Another key factor of bacterial division machinary, togheter with FtsZ, is FtsA. In this case the two-hybrid assay showed that the co-transformed strain containing *B. cenocepacia* FtsZ and FtsA has a β-galactosidase activity five-fold higher than the control strain (Figure 5A,B). This result indicates that a physical interaction occurs between the N-terminal domains of FtsA and FtsZ.

Among the early proteins involved in first steps of cell division, ZipA has been described in other bacteria to help the theatering of FtsZ to the membrane [57]. In this case, results indicate that *B. cenocepacia* FtsZ interacts with the C-terminus of ZipA, since the β-galactosidase activity value obtained from the two hybrid assay is 4.5 fold higher than the control (Figure 5A,B).

The last FtsZ-associated protein in *E. coli* that was analyzed is ZapA. The two-hybrid assay showed that the BTH101 strain carrying the vectors with *B. cenocepacia* FtsZ and ZapA has a β-galactosidase activity ten-fold higher than the control strain (Figure 5A,B), demonstrating the interaction of ZapA with the C-terminus of FtsZ.

Regarding the late proteins of cell division, the interaction between FtsZ and FtsE, FtsQ, FtsI and FtsN was evaluated. Results showed that the BTH101 clone expressing FtsZ and FtsE has a β-galactosidase activity fifty-fold higher than the control. This result demonstrates a physical interaction between FtsZ and the N-terminal region of FtsE in vivo. Moreover, the BACTH assay showed that the BTH101 strain transformed with both the vectors carrying FtsZ and FtsQ had a β-galactosidase activity sixteen-fold higher than the control strain. This result demonstrates a newly identified interaction between the C-terminal part of FtsZ and the N-terminal part of FtsQ, a key-player of the divisome reported to connect the early and the late proteins of cell division in *E. coli* [58]. In the search for other FtsZ interactors among the late components of cell division, two-hybrid assays were performed on clones expressing FtsZ together with FtsI or FtsN. In these cases, clones did not show any significative β-galactosidase activity (Figure 5A,B).

After the discovery that *B. cenocepacia* FtsZ interacts with both FtsA and ZipA, the physical interaction between FtsA and ZipA, previously described in *E. coli* [59] was investigated using the BACTH assay. The experiment revealed that the clone expressing FtsA and ZipA had a β-galactosidase activity thirty two-fold higher than the control strain. In *E. coli* FtsA interacts also with FtsN to connect early and late proteins [60]. In this work, we show that *B. cenocepacia* clones with FtsA and FtsN have a β-galactosidase activity five fold-higher than the control (Figure 5A,B). These results revealed that FtsA is able to interact with the N-terminal part of ZipA and that FtsA and FtsN interact each other with their C-termini. The interaction between FtsA and FtsQ was also tested, revealing that the early division protein FtsA of *B. cenocepacia* interacts also with the late protein FtsQ (Figure 5A,B), as previously described in *E. coli* [58].

#### 2.2.2. SulA Blocks FtsZ Polymerization In Vitro

**Figure 6:**
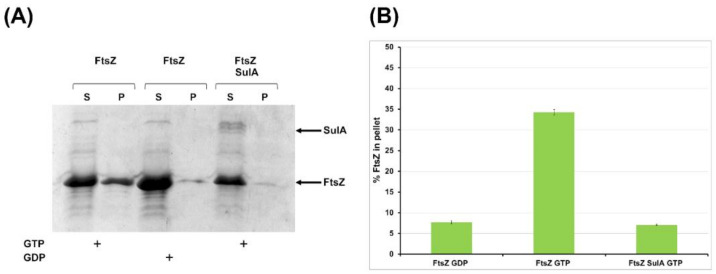
Effect of *B. cenocepacia* SulA on FtsZ in vitro polymerization. FtsZ was induced to polymerize in the presence or absence of SulA. (**A**) The polymeric FtsZ was collected by sedimentation and the amount of FtsZ in the pellets (P) was estimated by coomassie-blue staining of the SDS-PAGE. S, supernatant. (**B**) The bars indicate the relative quantification of FtsZ percentage in the pellet obtained by densitometry analysis. Data are the average ± SD of three independent experiments.

The BACTH assay highlighted the in vivo interaction of FtsZ and the putative SulA protein of *B. cenocepacia*. To confirm this physical interaction and its role in cell division, we expressed and purified *B. cenocepacia* SulA and carried out a co-sedimentation assay to check its effect on FtsZ polymerization. The co-sedimentation assay was performed in the absence and in the presence of SulA. In the presence of its co-factor GTP, FtsZ alone is able to polymerize, allowing the recovery of a good amount of protein in the pellet fraction (Figure 6A,B).

When SulA was added to the polymerization assay, in an equimolar concentration of FtsZ and in the presence of GTP to start the reaction, FtsZ was recovered only in the supernatant fraction, indicating that it was not able to polymerize. As a control, FtsZ in the presence of GDP (that could not polymerize) was recovered only in the supernatant (Figure 6A,B). These results demonstrated that the putative SulA of *B. cenocepacia* blocks FtsZ polymerization in vitro. Hence, it is possible to ascribe to this protein the role of regulator of FtsZ activity, as previously described in *E. coli*, where it blocks FtsZ activity in case of DNA damage during cell division [61].

#### 2.2.3. FtsZ and FtsA In Vitro Interaction

**Figure 7:**
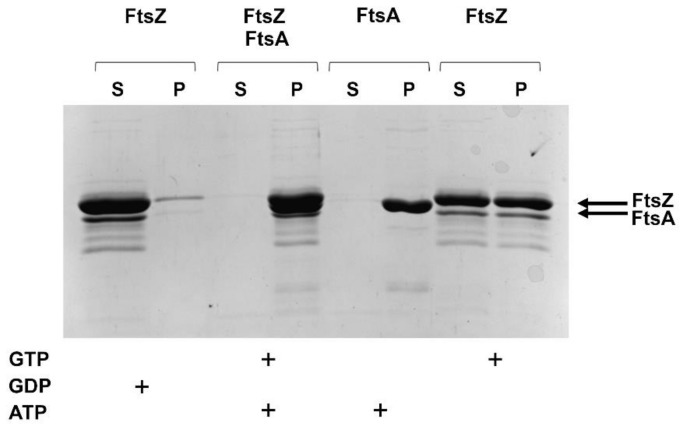
In vitro reconstitution of the FtsZ/FtsA interaction. FtsZ was induced to polymerize in the presence or absence of FtsA and GTP or GDP. The co-sedimentation was carried out in the presence of vesicles. The polymeric FtsZ and FtsA were collected by sedimentation and the amount of proteins in the pellet (P) was estimated by coomassie-blue staining of SDS-PAGE. S, supernatant.

FtsA and FtsZ interact in vivo as shown by the BACTH assay. To characterize this interaction in vitro, the *B. cenocepacia* FtsA was expressed, purified and used for co-precipitation assays in the presence of FtsZ and of vesicles that allow FtsA to interact with the membranes via its C-terminal amphipathic helix [62]. The liposomes co-sedimentation showed that the presence of lipids did not affect FtsZ polymerization, being about the 50% of the protein in the pellet fraction (Figure 7).

FtsA polymerized completely in the presence of ATP and vescicles (Figure 7), while in absence of ATP the protein was recovered only in the supernatant (data not shown). In the presence of both FtsZ and FtsA and their cofactors (GTP and ATP), all proteins were recovered in the pellet fraction (Figure 7). This result indicated that FtsA interaction with FtsZ increased the sedimentation of FtsZ in the pellet.

#### 2.2.4. FtsZ Interacts with ZipA In Vitro

**Figure 8:**
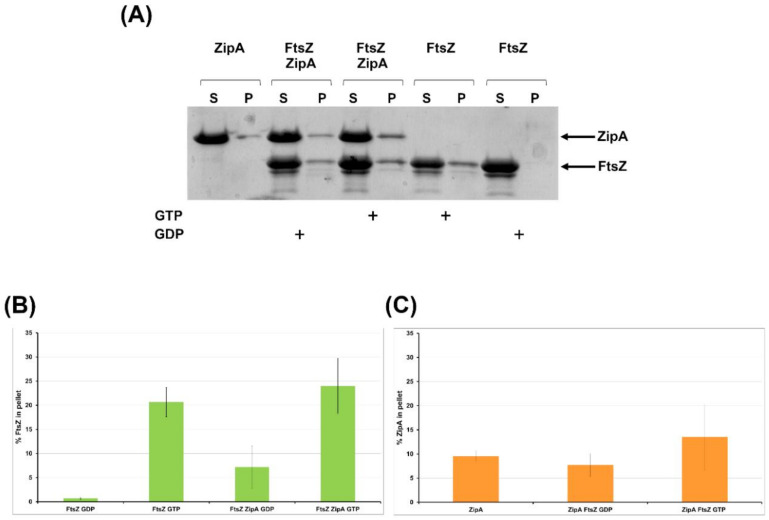
In vitro reconstitution of the FtsZ/ZipA interaction. FtsZ was polymerized in the presence or in the absence of ZipA with GTP or GDP. (**A**) The polymeric FtsZ was collected by sedimentation and the amount of FtsZ in the pellets (P) was estimated by coomassie-blue staining of the SDS-PAGE. S, supernatant. (**B**) and (**C**) The bars indicate the relative quantification of FtsZ (**B**) and ZipA (**C**) percentage in the pellet obtained by densitometry analysis. Data are the average ±SD of three independent experiments.

To confirm the physical interaction between *B. cenocepacia* FtsZ and ZipA, a co-sedimentation assay was set up (Figure 8A–C).

As reported in Figure 8, in the presence of ZipA and GTP the quantity of the polymerized FtsZ increased. Moreover, in the presence of ZipA and GDP, when the polymerization is not induced, FtsZ is present in the polymerized fraction together with ZipA (Figure 8A–C). In the presence of FtsZ and GTP, the quantity of ZipA in the polymerized fraction increased (Figure 8A–C). These results confirmed that *B. cenocepacia* ZipA interacts with FtsZ in its monomeric and filamentous forms.

## 3. Discussion

Cystic Fibrosis (CF) patients suffer from persistent lung infections throughout life and, among them, those caused by *B. cenocepacia* are the most dangerous. The accelerated deterioration of lung functions [39] and the significant increase of the mortality rate after lung transplantation, which remains the last option for patients with end-stage CF [63], make this pathogen a serious threat. For this reason, the characterization of new molecules active against this microorganism, as the C109 compound [46], is fundamental to expand the therapeutic options available, given its resistance to nearly every antibiotic currently used in clinics. However, the importance of the discovery of the C109 is not exclusively due to its potent antimicrobial activity, but also to the identification of its molecular target, the division protein FtsZ [47]. Indeed, this demonstrated the potential of the essential division proteins as druggable targets for a target-based drug design approach against *B. cenocepacia*, paving the way to the present in-depth study of the genetics and the protein interactions regulating the division process of this poorly characterized but very complex bacterium.

Starting from the identification of the *dcw* operon within the multireplicon genome of *B. cenocepacia*, we localized it on the chromosome 1, encompassing 17 tightly packed genes that reflect the conserved genetic arrangement of this cluster found in even very phylogenetically distant bacilli [26]. This gene order conservation across genera is known to be very rare [64]. However, in both Gram-positive and Gram-negative rod-shaped bacteria, the *dcw* gene arrangement is essential for the maintenance of the shape [26,65]. Indeed, this trait clearly undergoes to a constant positive selection absent for the other known shapes, as cocci, that probably have arisen after a consistent *dcw* gene order rearrangement [66]. In bacilli, the ordered clustering of *fts* and *mur* genes, involved in septation and peptidoglycan synthesis, gives an advantage to the synthesis of long transcriptional units. According to the genomic channeling theory proposed by Mingorance et al. [67], this can facilitate the compartmentalization of their translation and so promote the ordered assembly of protein complexes specifically required at the division site. The transcription of the *dcw* operon in *E. coli* and *B. subtilis*, the best characterized bacilli, is indeed organized in many long polycistronic mRNAs [29,68]. The presence of a transcript containing all the 16 genes was postulated in *E. coli*, observing that the transcription of the distal genes was greatly influenced by the activity of the promoters localized at the 5’ of the cluster [27,69]. Here we demonstrated the presence of an operon-wide transcript in *B. cenocepacia*, showing that the transcription can proceed from *mraZ* to *ftsZ*, giving rise to a long mRNA including 15 of the 17 *dcw* genes.

The transcription was controlled only by the active promoter here characterized, P_mra_Bc, localized in the non-coding region upstream of the first gene of the *dcw* operon, like its analogue P_mra_ in *E. coli* [53]. Accordingly, the transcription start site was experimentally identified at the 3’ of the P_mra_Bc sequence. Instead, the only terminator site identified was localized downstream of the last gene *lpxC*, as already reported in *E. coli* [70].

The presence of shorter transcription units was considered unlikely because of the lack of characterized or predicted promoters and terminator sites within the entire operon sequence. It is widely known that the timing of expression and the cytoplasmic concentration of FtsZ must be accurately controlled because it strongly influences the cell septation [65]. This regulatory function is usually carried out by promoters and regulatory sequences localized upstream of the *ftsA*-*ftsZ* region, as described in *E. coli* [54], *B. subtilis* [29], and *Bacillus mycoides* [31]. Intriguingly, in *B. cenocepacia* the distal region of the operon is characterized by the absence of active promoters, suggesting the presence of post-transcriptional control mechanisms involved in the modulation of the expression of *ftsZ*. Regarding the presence of a regulator of the transcription starting from P_mra_Bc, we demonstrated that in *B. cenocepacia* the protein MraZ is involved in this process, as already shown in *E. coli*, *Mycoplasma gallisepticum*, and *Corynebacterium glutamicum* [35,36,71]. In our experiments, the protein showed a strong binding affinity for a specific DNA fragment containing a series of four repeats of seven nucleotides. Each repeated sequence is probably recognized by MraZ as an octameric toroidal complex, as demonstrated in Mollicutes by Fisunov et al. [36], forming high molecular complexes with DNA.

After an accurate analysis of the *dcw* operon of *B. cenocepacia*, we focused our attention on the characterization of protein-protein interactions of the cell division machinery components. The cell division proteins of *B. cenocepacia* were selected based on the sequence homology with the best characterized cell division interactomes of *E. coli*. Proteins putatively involved in cell division have an intermediate level of conservation with the proteins of *E. coli*. By using the BACTH system, the interactions between the *B. cenocepacia* FtsZ and each protein selected was evaluated. The BACTH assay revealed that FtsZ monomers interact with each other with a head to tail interaction. This result confirmed what previously reported in other bacteria: FtsZ needs this interaction to complete the GTPase active site that allows protein polymerization [55].

Regarding the proteins that control cell division, SulA, described in *E. coli* and in other Gram-negative bacteria [56,61,72] blocks FtsZ activity in case of DNA damage. The results obtained using the BACTH system showed that the interaction between FtsZ and the putative SulA homologous (pBCA006) of *B. cenocepacia* occurs between their N-terminal parts. Moreover, using a co-sedimentation assay, we demonstrated that the putative SulA blocks FtsZ polymerization in vitro, confirming its role as FtsZ regulator also in *B. cenocepacia*.

Another key-player of the first steps of cell division is FtsA, for which the interaction with FtsZ has been experimentally described through many different approaches in *E. coli* [73]. As reported in the results, using the BACTH system we first reported that *B. cenocepacia* FtsA interacts with its N-terminal part with FtsZ in vivo. To confirm this interaction, an in vitro-sedimentation assay was carried out in the presence of vesicles. The results showed that *B. cenocepacia* FtsA is able to polymerize in the presence of ATP and vesicles and, when FtsZ and GTP were added to the reaction, FtsA increased the quantity of FtsZ recovered in the pellet fraction. These data suggest that FtsA polymers tether FtsZ to the membrane with a higher affinity compared to the previously described FtsA of *E. coli*, where FtsZ is not completely recovered in the pellet in the presence of FtsA [62,74]. Further mutational studies and pull-down experiments will clarify the FtsA behavior in *B. cenocepacia*.

In *E. coli* the early protein ZipA is one of the first protein interacting with FtsZ. Results obtained from the BACTH assay showed that ZipA of *B. cenocepacia* interacts with the C-terminal part of FtsZ, similarly to what previously obtained in *E. coli* [75]. Furthermore, the interaction between FtsZ and ZipA was evaluated through the in vitro sedimentation assay, showing that ZipA tethers FtsZ in the pellet fraction also when the polymerization is not induced. These results confirmed the in vitro interaction between FtsZ and ZipA of *B. cenocepacia*, as previously reported in *E. coli* [57].

Looking at the late proteins involved in cell division, FtsE, a member of the ABC transporter superfamily, has an important role in the connection of the Z ring with the late division proteins and in the initiation of the bacterial cell constriction [76,77]. Here, a physical in vivo interaction between the N-terminal region of FtsE and the C-terminal region of FtsZ of *B. cenocepacia* was validated. This type of interaction was previously described in *E. coli* using an in vitro pull-down experiment [78] and the BACTH system [10].

In *E. coli* FtsQ acts as a sensing mechanism that promotes the passage from early to late phases of cell division [58]. Using the bacterial two hybrid assay, we demonstrate that FtsQ interacts directly with FtsZ in *B. cenocepacia*, while in *E. coli* FtsQ interacts with different components of cell division, such as FtsI, FtsN, FtsW and FtsK [79].

On the other hand, the BACTH assay revealed no other interactions between FtsZ and the components of the late phase of cell division FtsI and FtsN as in *E. coli*.

Although it has been demonstrated that there is an order of the division protein recruitment, there are evidences in *E. coli* that these proteins interact each other outside of the division process [80]. Using two-hybrid experiments, we demonstrated that in *B. cenocepacia* the scenario is similar: indeed, FtsA is able to interact using its C-terminal part with both ZipA and FtsN, two proteins involved in different phases of cell division.

The cell division machinery is a well conserved biological system in bacteria, but there are some peculiarities for each bacterial species, as previously described in *Neisseria gonorrhoeae* in which, for example, ZipA does not interact with the other components of the divisome and FtsA interacts with FtsW [81]. For instance, in this work we described for the first time the interaction between FtsZ and FtsQ. All these results pave the way to the identification of new drug target candidates in the high multi-drug resistant *B. cenocepacia*, which will be used to search for novel compounds able to inhibit bacterial growth.

## 4. Materials and Methods

### 4.1. Bacterial Strains, Plasmids, and Culture Conditions

The bacterial strains and plasmids used in this work are listed in Table S4. *E. coli* and *B. cenocepacia* cultures were growth in Luria-Bertani (LB) broth (Difco, BD, Sparks, MD, USA) with shaking at 200 rpm at 37 °C or in LB agar plate at 37 °C. *E. coli* culture for BACTH complementation assay was grown in LB with shaking at 200 rpm at 30 °C. The *E. coli* XL1-Blue strain was used in all of the cloning steps of the BACTH system. BACTH complementation assays were carried out with the *E. coli cya* strain BTH101 (Table S4).

For plasmid selection and maintenance, the following antibiotics were used: Kanamycin (Applichem, Panreac, Darmstadt, Germany) 50 μg/mL (*E. coli*); Gentamicin (Sigma, St. Louis, MO, USA) 20 μg/mL (*E. coli*), 500 μg/mL (*B. cenocepacia*); Ampicillin (Applichem, Panreac, Darmstadt, Germany) 100 μg/mL (*E. coli*), Chloramphenicol (Sigma) 34 μg/mL (*E. coli*), Streptomycin (Sigma) 100 μg/mL (*E. coli*). During the BACTH complementation assays, media were supplemented with 0.5 mM isopropyl β-D-thiogalactopyranoside (IPTG) and 40 μg/mL 5-Bromo-4-chloro-3-indolyl-β-D-galactopyranoside (X-gal).

### 4.2. B. cenocepacia J2315 RNA Extraction and RT-PCR

The total RNA of *B. cenocepacia* J2315 was extracted following the protocol of the Direct-zol RNA Miniprep Kit (Zymo Research, Irvine, CA, USA) but adding a preliminary step in order to improve the cell lysis efficiency: bacterial cells, resuspended in TRI Reagent^®^ (Zymo Research) and mixed with zirconia beads (Ambion, Austin, TX, USA) in a 2 mL bead beating tube, were subjected to 2 cycles of 4 min at the maximum speed of bead beating using the Minilys personal homogenizer (Montigny-le-Bretonneux, France). The obtained RNA was treated twice with the DNA-free™ DNA Removal Kit (Invitrogen, Carlsbad, CA, USA) to completely remove DNA contaminations. After that, the RNA was retrotranscribed following the ProtoScript^®^ II Reverse Transcriptase (NEB, Ipswich, MA, USA) protocol. cDNAs were then used as templates to amplify by PCR specific fragments using primers annealing on two consecutive *dcw* cluster genes. All the primers used for reverse transcription PCR and cDNA PCR are listed in Table S1. The amplicons obtained were purified and sequenced and the specificity of the results was validated using PCR negative controls (using RNA as template for the cDNA amplification) and positive controls (amplification of the genomic DNA using the cDNA primers).

### 4.3. Promoter Activity Assessment by β-Galactosidase Activity Assay

The *dcw* promoter activity of *B. cenocepacia* J2315 were measured by β-galactosidase activity assay taking advantage of the pSU11 vector [82] containing transcriptional *lacZ* fusions. First, two overlapping fragments, containing the putative *dcw* promoter, were amplified by PCR from the intergenic region upstream of the gene *mraZ*, using dcwPROMOrev2 coupled with dcwPROMOfor1_2 or dcwPROMOfor2_2 primers (Table S2). Additional four fragments, covering the entire *ddl*-*ftsA* region, were amplified by PCR to check the presence of active promoters in the distal part of the operon by using the primers murCPROMOfor1-ddlPROMOrev1, ftsQPROMOfor1-ftsQPROMOrev1, ftsAPROMOfor1-ftsAPROMOrev1, ftsAPROMOfor2-ftsAPROMOrev2 (Table S2). The purified fragments were cloned upstream of the promoterless *lacZ* reporter gene into the pSU11 vector, previously digested with *Hind*III and dephosphorylated using alkaline phosphatase (Roche, Basel, Switzerland), following the In-fusion HD Cloning Kit protocol (Takara, Shiga, Japan). The resulting plasmids (Table S3) were transferred from *E. coli* to *B. cenocepacia* K56-2 by triparental mating. The β-galactosidase assay was carried out as described before [83] with minor changes. Bacterial cultures were grown overnight in liquid LB, diluted 1/50 in fresh LB and recultured for six hours. Then, the precise OD_600_ were measured and cells from 1 mL of culture were harvested by centrifugation, resuspended in the same volume of Z-buffer (60 mM Na_2_HPO_4_, 40 mM NaH_2_PO_4_ pH = 7, 10 mM KCl, 1 mM MgSO_4_, 50 mM DTT) and permeabilized with 25 µL of chloroform and 25 µL of 0.1% SDS. The bacterial suspensions were vortexed for 10 s and 500 µL of each were diluted 1/2 in Z-buffer and incubated at 28 °C for 10 min. 200 µL of o-nitrophenyl-β-D-galactoside (ONPG) solution (4 mg/mL in Z-buffer) were added to each sample to start the reaction; then the samples were vortexed, and further incubated at 28 °C. When the samples turned yellow, the reactions were stopped adding 500 µL of 1 M Na_2_CO_3_. Before the spectrophotometric quantification, samples were centrifuged at 15,000× *g* for 10 min at room temperature and finally 1 mL of cell debris-free supernatant was used to measure the absorbance at 420 nm and 550 nm, allowing the calculation of the promoter activity (Miller Units).

### 4.4. 5′-Rapid Amplification of cDNA Ends (5′-RACE)

For the identification of the *dcw* operon transcription start site in *B. cenocepacia* J2315, 5’ rapid amplification of cDNA ends (5’-RACE) was performed. The RNA extraction, DNase treatment and reverse transcription were carried out as mentioned above, using the primer mraZRACErev1 (Table S2) for the RT-PCR. Subsequently, to degrade the RNA of the resulting cDNA-RNA heteroduplex and improve the further step yield, the sample was treated with ribonuclease H (Promega, Madison, WI, USA) adding 2 U of enzyme directly in 20 uL of RT-PCR mix after the reverse transcription reaction and incubating at 37 °C for 30 min. Then, the cDNA was purified and a tail of polyadenosine was added to the 3’ end by terminal deoxynucleotidyl transferase (Promega) reaction, according to the manufacturer’s instructions. The cDNA was amplified by PCR using the polyT universal forward primer RA1 (Table S2) coupled with the reverse primer mraZRACErev2 (Table S2). The reaction product of the first PCR was used as template for the nested PCR performed with the primers RA2 and mraZRACErev3 (Table S2), designed to amplify a fragment within the first product sequence. The resulting amplified fragment was sequenced to identify the *dcw* cluster transcription start site.

### 4.5. Cloning, Expression, and Purification of B. cenocepacia MraZ

The gene *mraZ* (*BCAL3471*) of *B. cenocepacia* J2315 was amplified from the genomic DNA by PCR using the primers mraZpET28aFOR and mraZpET28aREV (Table S2). The two primers were designed following the In-fusion HD Cloning Kit protocol instructions (Takara) and the PreScission protease (GE Healthcare, Chicago, IL, USA) cleavage site was added to the forward primer to remove the 6-histidine tag from the protein. The purified fragment was cloned into the linearized pET28a (Merck Millipore, Burlington, MA, USA) vector, digested with *Bam*HI/*Hind*III restriction enzymes, by recombination according to the In-fusion HD Cloning Kit protocol, obtaining the pET28a-MraZ (Table S3). To express the protein MraZ, *E. coli* BL21(DE3) strain was used. 3L of LB supplemented with Kanamycin were inoculated 1/50 with the overnight grown starter culture and incubated at 37 °C with shaking until OD_600_ = 0.6 was reached. At this point, the protein expression was induced with 0.5 mM of IPTG and the culture was incubated overnight at 18 °C. The cells were harvested by centrifugation, resuspended in buffer A (25 mM Tris-HCl pH = 7.5, 300 mM NaCl, 5 mM MgCl_2_, glycerol 5%), supplemented with 1 mM of the non-specific protease inhibitor Phenylmethanesulfonyl fluoride (PMSF) and lysed by sonication. The lysate was centrifuged at 50,000× *g* for 30 min and the protein was purified from the cell-free extract using immobilized metal affinity chromatography (IMAC) on a HiTrap TALON crude column (1 mL, GE Healthcare). The purified protein was dialyzed against the buffer B (50 mM Tris-HCl pH = 7.5, 300 mM NaCl, 5 mM MgCl_2_, glycerol 5%, 1 mM Dithiothreitol (DTT)) and digested with PreScission protease (GE Healthcare) to remove the 6-histidine tag. Finally, the protein MraZ was further purified in the same buffer on a nickel nitrilotriacetic acid resin (Ni-NTA, Qiagen, Hilden, Germany) packed in a column, quantified, and stored at −80 °C.

### 4.6. Electrophoretic Mobility Shift Assay (EMSA)

To characterize the binding site of the transcriptional regulator MraZ, electrophoretic mobility shift assay (EMSA) was used. The non-coding region upstream of *mraZ*, containing the putative binding site, was amplified by PCR using mraZBS1for, mraZBS1rev and mraZBS2for, mraZBS2rev primers (Table S2). These primers were labeled with 6-carboxyfluorescein to be easily detectable on the gel using the ChemiDoc XRS+ System (Bio-Rad, Milano, Italia) and were designed to amplify the two DNA probes that consisted in two overlapping fragments of 250 bp covering the whole intergenic region. The fragments were named BS1-6-Fam and BS2-6-Fam and their sequences were confirmed by sequencing. 0.34 pmoles of the labeled DNA probes were incubated with increasing quantities of MraZ (250, 300, 350, 400 pmoles) in a reaction volume of 25 µL in binding buffer (20 mM Tris-HCl pH = 7.5, 100 mM NaCl, 5 mM MgCl_2_, 0.1 mM DTT, 150 ng/µL BSA, 50 ng/µL deoxyribonucleic acid from herring sperm) for 30 min at 37 °C and loaded on an agarose gel 1.5%. The electrophoretic run was performed in TBE 0.5× for 1 h at 100 V at 4 °C in the dark, after an initial equilibration of the gel for 30 min at 60 V in TBE 0.5×. The gel was then visualized using the ChemiDoc XRS+ System (Bio-Rad). The specificity of the binding was verified repeating the same experiment adding a 20× excess of specific (non-labeled BS2 fragment) or non-specific (non-labeled 250 bp fragment amplified by PCR from the gene *cepI* of *B. cenocepacia* J2315 with mraZBSNSfor and mraZBSNSrev (Table S2) primers), DNA probe competitors.

### 4.7. Construction of the Recombinant Plasmid of the BACTH Complementation Assays

The genes coding for FtsZ, FtsA, FtsE, FtsQ, FtsI, FtsN, ZipA, ZapA and SulA were amplified by using the appropriate primers listed in Table S5 and the genomic DNA of *B. cenocepacia* J2315 as template. The PCR products were inserted by recombination, following the In-fusion HD Cloning Kit protocol instruction (Takara), into the plasmids of the BACTH system (Euromedex, Souffelweyersheim, France) pUT18, pUT18C, pKT25 and pKNT25, previously digested with the corresponding restriction enzymes, obtaining the vectors listed in Table S3. The resulting recombinant plasmids expressed hybrid proteins in which the polypeptides of interest were fused to the C- or N-termini of the T25 and T18 fragments of adenylate cyclase, respectively.

### 4.8. BACTH Complementation Assays

For BACTH complementation assays, recombinant pUT18, pUT18C, pKT25 and pKNT25 carrying the selected genes were used in various combinations to cotransform BTH101 cells. The transformants were plated onto LB-IPTG-XGal medium containing Kanamycin and Ampicillin and incubated at 30 °C for 48 h. Efficiencies of interactions between different hybrid proteins were evaluated in solid media (LB and MacConkey) and in liquid culture quantifying the β-galactosidase activity. The β-galactosidase assays were performed as described previously, on strains grown in LB broth in the presence of 0.5 mM IPTG, Ampicillin and Kanamycin at 30 °C for 18 h. A level of β-galactosidase activity at least four-fold higher than that measured for BTH101 (pUT18/pKT25) cells was considered to indicate an interaction.

### 4.9. Cloning, Expression, and Purification of pBCA006, the Putative B. cenocepacia SulA

The protein pBCA006 of *B. cenocepacia* J2315, the putative SulA, was expressed using the following protocol. The gene *pBCA006* was amplified from genomic DNA by PCR using the primers sulApBADM41for and sulApBADM41rev (Table S2). The PCR product was inserted by recombination, following the In-fusion HD Cloning Kit protocol instruction (Takara), into the pBADM-41 plasmid, previously digested with *Nco*I/*Eco*RI, obtaining the pBADM-41-SulA (Table S3). This plasmid was co-transformed by electroporation with the pG-Tf2 vector (Chaperone plasmid set, Takara), expressing the *E. coli* chaperones GroEL-GroES and tig upon induction, into *E. coli* TOP10 competent cells. The consequent inducible overexpression of the GroEL-ES complex in this strain assisted the correct folding of MBP-SulA, increasing the quantity of soluble protein produced, which otherwise was completely accumulated within inclusion bodies given the intrinsic instability of SulA. The cells were grown in 3 L of LB supplemented with Ampicillin and Chloramphenicol, and 5 ng/mL of Tetracycline to induce the chaperones expression, until OD_600_ = 0.6 was reached, then the protein expression was induced with 0.2% of L-arabinose overnight at 18 °C. The cells were collected by centrifugation, resuspended in buffer C (50 mM Tris-HCl pH = 7, 5 mM MgCl_2_, glycerol 5%) supplemented with 1 mM of PMSF and lysed by sonication. Subsequently, the lysate was centrifuged at 50,000× *g* for 30 min and a solution of ATP pH = 7 was added to the supernatant obtaining a final concentration of 5 mM, in order to facilitate the detachment of GroEL from MBP-SulA. After 15 min of incubation at 4 °C, the extract was loaded on a MBPTrap HP (5 mL, GE Healthcare), eluting MBP-SulA complexed with GroEL with 10 mM of maltose in buffer C. In order to eliminate the chaperone contamination, the eluate was further purified using cation exchange chromatography. A column of SP Sepharose Fast Flow resin (GE Healthcare) was used and the proteins were eluted in buffer C with a (0–1 M) KCl gradient. The fractions containing exclusively MBP-SulA were merged, desalted using a HiPrep 26/10 Desalting column (GE Healthcare), and eluted in buffer D (20 mM Tris-HCl pH = 7.9, 50 mM KCl, 1 mM EDTA, glycerol 10%). Finally, the protein was quantified and stored at −80 °C.

### 4.10. Cloning, Expression, and Purification of B. cenocepacia FtsA

To express the protein FtsA of *B. cenocepacia* J2315, the gene *ftsA* (*BCAL3458*) was amplified by PCR, using the primers ftsASUMOfor and ftsASUMOrev (Table S2), with genomic DNA as template. The fragment obtained was purified and inserted into the pETSUMO (Invitrogen) plasmid, using the In-fusion HD Cloning Kit protocol (Takara), and then the pETSUMO-FtsA (Table S3) was transformed into *E. coli* BL21(DE3) competent cells by electroporation. The culture was grown in LB supplemented with Kanamycin and the protein production was induced with 0.5 mM of IPTG overnight at 20 °C. The cells were harvested by centrifugation, resuspended in buffer E (50 mM Tris-HCl pH = 7.5, 300 mM NaCl, 5 mM MgCl_2_, glycerol 5%) containing 1 mM of PMSF and lysed by sonication. The lysate was clarified by centrifugation at 50,000× *g* for 30 min and the supernatant was applied on a HisTrap HP nickel column (1 mL, GE Healthcare), eluting the protein with 250 mM imidazole in buffer E. Then, the purified protein was dialyzed overnight against buffer B and the SUMO protease was used to remove the SUMO protein. A further purification step was carried out by size exclusion chromatography, using a HiLoad 16/60 Superdex-75 column (GE Healthcare) in buffer D, obtaining the purified FtsA that was concentrated to 5 mg/mL and stored at −80 °C.

### 4.11. Cloning, Expression, and Purification of B. cenocepacia ZipA

To express the soluble fragment of the protein ZipA of *B. cenocepacia* J2315, the gene *zipA* (*BCAL2097*) was amplified by PCR, using the primers ZipASUMOFor and ZipASUMOrev (Table S2), with genomic DNA as template. The fragment obtained was purified and inserted into the pETSUMO (Invitrogen) plasmid, using the In-fusion HD Cloning Kit protocol (Takara), and then the pETSUMO-ZipA (Table S3) was transformed into *E. coli* BL21(DE3) competent cells by electroporation. The culture was grown in LB supplemented with Kanamycin and the protein production was induced with 0.5 mM of IPTG overnight at 18 °C. The cells were harvested by centrifugation, resuspended in buffer E containing 1 mM of PMSF and lysed by sonication. The lysate was clarified by centrifugation at 50,000× *g* for 30 min and the supernatant was applied on a HisTrap HP nickel column (1 mL, GE Healthcare), eluting the protein with 250 mM imidazole in buffer E. Then, the purified protein was dialyzed overnight against buffer B and the SUMO protease was used to remove the SUMO protein. A further purification step was carried out by size exclusion chromatography, using a HiLoad 16/60 Superdex-75 column (GE Healthcare) in buffer D, obtaining the purified ZipA that was concentrated to 10 mg/mL and stored at −80 °C.

### 4.12. Co-Sedimentation Assays of FtsZ in the Presence of ZipA or SulA

The reaction mixtures containing 25 mM PIPES (pH 6.8), 10 mM MgCl_2_, 12 µM FtsZ, 12 µM ZipA or SulA and 2 mM GTP or GDP were incubated for 10 min at 30 °C and 300 rpm to allow the polymerization to occur. Samples were then ultracentrifuged at 350,000× *g* for 10 min at 25 °C, and the supernatant was immediately separated from the pellet, which contains the protein polymers. The samples were analyzed by SDS-PAGE on 12% polyacrylamide gels. The quantification of FtsZ polymerization was performed as previously described [84], using densitometry by calculating the percentage of the relative intensity of the bands.

### 4.13. Co-Sedimentation Assays of FtsZ in the Presence of FtsA and Vesicles

Vesicles were prepared using *E. coli* total lipid extract (Avanti Polar Lipids , Alabaster, AL, USA) in 25 mM PIPES (pH 6.8), 300 mM KCl and 10 mM MgCl_2_ using sonication. The reaction mixtures contained 2 mg/mL of vesicles with pre-spun proteins at a final concentration of 12 µM, 25 mM PIPES (pH 6.8), 10 mM MgCl_2_, and 2 mM GTP, GDP or ATP. The reactions were incubated for 10 min at 30 °C and 300 rpm to allow the polymerization to occur, then samples were centrifuged at 20,000× *g* at 20 °C for 25 min. The supernatants were immediately removed for analysis, the pellets resuspended with 25 mM PIPES (pH 6.8), 300 mM KCl and 10 mM MgCl_2_ (same volume as the supernatants) and solubilized with SDS gel loading buffer. Samples were analysed by SDS–PAGE on 12% polyacrylamide gels.

## 5. Conclusions

The importance of cell division as a new source of drug targets deserved a careful description in the multi-drug resistant bacterium *Burkholderia cenocepacia*, for which a lack of knowledge was evident. In this work we described the organization of the division cell wall (*dcw*) cluster, in which 15 genes can be transcribed as a polycistronic mRNA from *mraZ* to *ftsZ*. We also highlighted that the transcription is under the control of a strong promoter regulated by MraZ. Moreover, the interaction of FtsZ with the other components of the divisome machinery was shown. Importantly, the direct interaction with FtsQ has never been reported before. We validated the role of SulA as FtsZ inhibitor, and the roles of FtsA and ZipA as tethers of FtsZ polymers. Our results lead to a deep knowledge of cell division in *B. cenocepacia*, paving the way for future antimicrobial design based on the divisome.

## Supplementary Materials

The following are available online at https://www.mdpi.com/2079-6382/9/12/841/s1, Figure S1: Representation of the distal genes of the *dcw* cluster. Table S1: List of primers used for the transcription analysis of the *dcw* operon, and expected length of the PCR fragments. Table S2: List of primers used for gene and promoter sequences cloning into plasmids, EMSA and 5’-RACE. Table S3: List of plasmids used in this work. Table S4: List of strains used in this work. Table S5: List of primers used for cloning the divisome genes into the BACTH plasmids.
