# Supplementary material for: Molecular Characterization of the Burkholderia cenocepacia dcw Operon and FtsZ Interactors as New Targets for Novel Antimicrobial Design
Source: Antibiotics (Basel). 2020 Nov 24;9(12):841. doi: 10.3390/antibiotics9120841 (PMC7761207; doi:10.3390/antibiotics9120841)
Supplement: Supplementary file 1 [file antibiotics-09-00841-s001.pdf]

antibiotics

# Molecular Characterization of the *Burkholderia cenocepacia dcw* Operon and FtsZ Interactors as New Targets for Novel Antimicrobial Design

**Gabriele Trespidi †, Viola Camilla Scoffone †, Giulia Barbieri, Giovanna Riccardi, Edda De Rossi * and Silvia Buroni ***

Department of Biology and Biotechnology "Lazzaro Spallanzani", University of Pavia, 27100 Pavia, Italy; gabriele.trespidi01@universitadipavia.it (G.T.); viola.scoffone@unipv.it (V.C.S.); giulia.barbieri@unipv.it (G.B.); giovanna.riccardi@unipv.it (G.R.)

* Correspondence: edda.derossi@unipv.it (E.D.R.); silvia.buroni@unipv.it (S.B.)

† These authors contributed equally to this work.

**Supplementary figures**

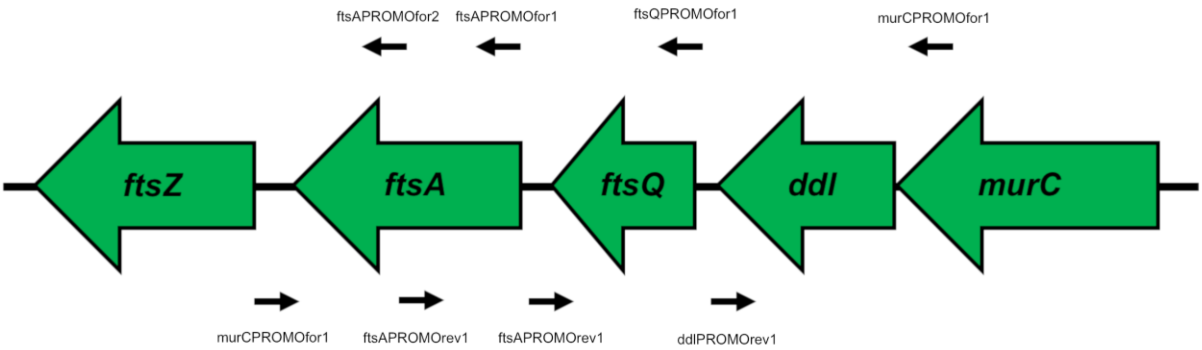


**Figure S1.** Representation of the distal genes of the *dcw* cluster. The black arrows represent the primers used for the amplification of the fragments tested for their promoter activity.

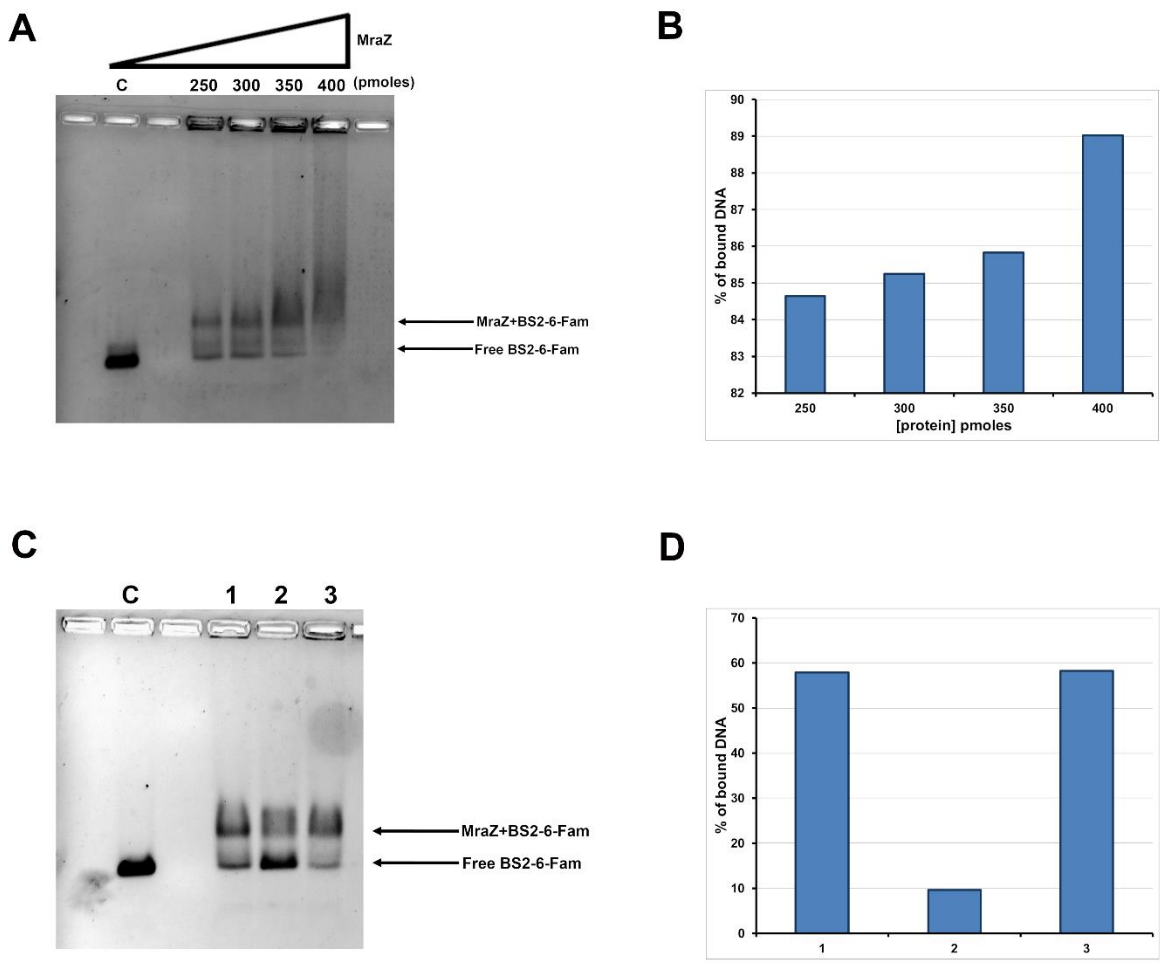


**Figure S2.** Electrophoretic mobility shift assay of the MraZ protein. (A) The gel represents the band shift using a constant BS2-6-Fam DNA amount (0.34 pmoles) and increasing quantities of MraZ. (B) The bars indicate the relative quantification of the percentage of the bound DNA obtained by densitometry analysis in function of the protein concentration. (C) Demonstration of the specificity of the binding of MraZ to the fragment BS2, using a fixed amount of protein (300 pmoles) and BS2-6-Fam (0.34 pmoles) (lane 1), a 20X excess of non-labeled BS2 fragment which competes for the binding of MraZ (lane 2) , or a 20X concentration of non-specific, non-labeled competitor (a 250 bp *cepI* fragment) (lane 3). (D) The bars indicate the relative quantification of the percentage of the bound DNA of the corresponding lanes obtained by densitometry analysis.

**Supplementary tables**

**Table S1**

| Primer | Sequence 5′- 3′ | Expected fragment |
|---|---|---|
| mraZRTfor1 | GTGTTCCAAGGGGCGTCGGC | 661bp |

| | | |
|---|---|---|
| mraWRTrev1 | GCATCCTTGATGCCCTGCGCCGTCT | |
| mraWRTfor1 | AAGTCGTACTAGACGCGGCA | 503bp |
| ftsLRTrev1 | TCGAGATCGGCTGCATTTTCAGCGA | |
| ftsLRTfor1 | GCCTCAATATCTTCCTGCTG | 509bp |
| ftsIRTrev1 | CCTGCTTCTGATAGAACGCG | |
| ftsIRTfor1 | GGGTAGCGGCGCAAAGCATA | 514bp |
| murERTrev1 | CGGGCATCCCGGTGCCGAGCGT | |
| murERTfor1 | GGCTCGAGTCGGTCAATGGC | 561bp |
| murFRTrev1 | ATCAGGCGGGCGGCTTCGCCG | |
| murFRTfor1 | AAGATGGAGCGCGTGGTCGA | 512bp |
| mraYRTrev1 | ACAGCCCGATCACCGATTGC | |
| mraYRTfor1 | ACCTGCTGTTCCCGCACATC | 571bp |
| murDRTrev1 | CCTCGCGGGTATCGGCAATA | |
| murDRTfor1 | TCGACATGTTCACGAACTAC | 529bp |
| ftsWRTrev1 | GTTCACGCCCTTGCCGACAT | |
| ftsWRTfor1 | ACGATGCTCGTGTGGCTGTC | 581bp |
| murGRTrev1 | GGTGCCGCCTGCCATCACCA | |
| murGRTfor1 | AGGCTACCGACGAAGTCGCG | 568bp |
| murCRTrev1 | GAACGACGCATCCGACTCGT | |
| murCRTfor1 | CTGGCGCTGGCGGAGTTCAA | 544bp |
| ddlRTrev1 | GAACAACACTGCCACCTTGC | |
| ddlRTfor1 | CGCGACGCACGACAAGATCG | 512bp |
| ftsQRTrev1 | CGCCAACACGAGCAGCAGCA | |
| ftsQRTfor1 | GGCAGGCATGCGGTTCCTGAC | 557bp |
| ftsARTrev1 | CCCGTCACGATGTGCACCTTC | |
| ftsARTfor1 | AAGACATCAAGGTCGGCTACGGGAT | 616bp |
| ftsZRTrev1 | TGGTCTCGGTTTCCAGCATT | |
| ftsZRTfor1 | GAAGAAGCAGCAGTCGGCAC | 581bp |
| BCAL3456RTrev1 | AATCCGAAGATCACCACCCG | |
| BCAL3456RTfor1 | CAAATTCGAAGTGAGCGATG | 573bp |

antibiotics MDPI

| | |
|---|---|
| lpxCRTrev1 | TCGACATACAGGTTGTCGAT |

**Table S1:** List of primers used for the transcription analysis of the *dcw* operon, and expected length of the PCR fragments.

**Table S2**

| Primer | Sequence 5′- 3′ | Restriction site |
|---|---|---|
| dcwPROMOfor1_2 | GGTATCGATAAGCTTACGCCCGTCTGGGCCGTCTT | *Hind*III |
| dcwPROMOrev2 | TCTAGCTAGAAGCTTTTTCCGCTCTCCCGTTCAGG | *Hind*III |
| dcwPROMOfor2_2 | GGTATCGATAAGCTTATTCGTCAAAAAAGCGGGCC | *Hind*III |
| murCPROMOfor1 | GGTATCGATAAGCTTCATTCAACAGAAGGCATGAC | *Hind*III |
| ddlPROMOrev1 | TCTAGCTAGAAGCTTACGTCGTTCCGTTCCTGCGT | *Hind*III |
| ftsQPROMOfor1 | GGTATCGATAAGCTTATGTGGAACAACGTTCGCCA | *Hind*III |
| ftsQPROMOrev1 | TCTAGCTAGAAGCTTAAAGTGCTCTTGCGTGTGAT | *Hind*III |
| ftsAPROMOfor1 | GGTATCGATAAGCTTATGAGCAAAGACTACAAGGA | *Hind*III |
| ftsAPROMOrev1 | TCTAGCTAGAAGCTTGATGTCGACCAGCACCACGC | *Hind*III |
| ftsAPROMOfor2 | GGTATCGATAAGCTTGGCGGCGGCACGACGGACAT | *Hind*III |
| ftsAPROMOrev2 | TCTAGCTAGAAGCTTTGTTGCCTCCGTCAAGAGAA | *Hind*III |
| mraZBS1for | GATTGGCGCCGGGGTGGCGT | |
| mraZBS1rev | GGAGCGGCCCGCTTTTTTGA | |
| mraZBS2for | AAGTTGCACTAGCTCATTCA | |
| mraZBS2rev | TTTCCGCTCTCCCGTTCAGG | |
| mraZpET28aFOR | ATGGGTCGCGGATCCCTGGAAGTTCTGTTCCAGGGGCCCaTGTTCCAAGGGGCGTCGGC | *Bam*HI |
| mraZpET28aREV | TGCGGCCGCAAGCTTTCAGAACGTGAAATTCTTCA | *Hind*III |
| sulApBADM41for | TTCAGGGCGCCATGGggCTGGAAGTTCTGTTCCAGGGGCCCCACCCCGCCCTCGCC | *Nco*I |
| sulApBADM41rev | ACGGAGCTCGAATTCTCAGGCGACGGCGCC | *Eco*RI |
| ftsASUMOfor | GAGAACAGATTGGTGGTATGAGCAAAGACTACAAGGA | |
| ftsASUMOrev | ATACCTAAGCTTGTCTTCAGAAGTTGCTCAGGAACC | |
| ZipASUMOFor | GAGAACAGATTGGTGGTTGGCAGGGCGCGAAAGTGCGGCGC | |

| | |
|---|---|
| ZipASUMORev | ATACCTAAGCTTGTCTTTACTGGCTGAAGAGGCG GCGCGTGAC |
| mraZBSNSfor | ACACGAACTCGCGGCGGATC |
| mraZBSNSrev | TTGCGGCAGCGGCATGTCCT |
| mraZRACErev1 | CATTCCCAACAACATGACTT |
| RA1 | GACCACGCGTATCGATGTCGAC(T)$_{16}$ |
| mraZRACErev2 | CGGAAACAGCAACAGGCAGC |
| RA2 | GACCACGCGTATCGATGTCGAC |
| mraZRACErev3 | GTCACAGTCACCCGTCCTTC |

**Table S2:** List of primers used for gene and promoter sequences cloning into plasmids, EMSA and 5′-RACE. The restriction site is underlined and the prescission protease sequence is in blue.

## Table S3

| Plasmid | Description | Source |
|---|---|---|
| pSU11 | *E. coli-Burkholderia* shuttle vector containing *lacZ* reporter gene downstream of the MCS, Gm$^r$ | Jenul *et al.*, 2018 |
| pSU11-161 | pSU11 containing the *dcw* predicted promoter sequence in a 161 bp fragment | This study |
| pSU11-289 | pSU11 containing the *dcw* predicted promoter sequence in a 289 bp fragment | This study |
| pSU11-ftsQp | pSU11 containing the DNA sequence of the gene *ddl* | This study |
| pSU11-ftsAp | pSU11 containing the DNA sequence of the gene *ftsQ* | This study |
| pSU11-ftsZp1 | pSU11 containing the first half of the *ftsA* gene sequence | This study |
| pSU11-ftsZp2 | pSU11 containing the second half of the *ftsA* gene sequence | This study |
| pET28a | Expression vector IPTG inducible, Kan$^r$ | Novagen |
| pET28a-MraZ | pET28a containing MraZ coding sequence | This study |
| pBADM-41 | Expression vector for toxic or unstable proteins, containing MBP fusion, controlled by *araBAD* promoter, Amp$^r$ | Laboratory collection |
| pBADM-41-SulA | pBADM-41 containing SulA coding sequence | This study |
| pETSUMO | Expression system incorporating a SUMO fusion, IPTG inducible, Kan$^r$ | Invitrogen |
| pETSUMO-FtsA | pETSUMO containing FtsA coding sequence | This study |
| pETSUMO-ZipA | pETSUMO containing ZipA coding sequence | This study |
| pUT18 | Derivative of pUC19. A MCS allows construction of in-frame fusions at the N-terminal end of the T18 polypeptide under the control of a lac promoter. | Karimova *et al.*, 1998 |

| | | |
|---|---|---|
| pUT18C | Derivative of pUC19. A MCS allows construction of in-frame fusions at the C-terminal end of the T18 polypeptide under the control of a lac promoter. | Karimova *et al.*, 1998 |
| pKT25 | Derivative of pSU40. A multicloning site sequence (MCS) allows construction of in-frame fusions at the N-terminal end of the T25 polypeptide under the control of a lac promoter. | Karimova *et al.*, 1998 |
| pKNT25 | Derivative of pSU40. A MCS allows construction of in-frame fusions at the C-terminal end of the T25 polypeptide under the control of a lac promoter. | Karimova *et al.*, 1998 |
| pUT18FtsZ | pUT18 containing the T18 fused to the C-terminal end of FtsZ | This study |
| pUT18CFtsZ | pUT18C containing the T18 fused to the N-terminal end of FtsZ | This study |
| pKT25FtsZ | pKT25 containing the T25 fused to the N-terminal end of FtsZ | This study |
| pKNT25FtsZ | pKNT25 containing the T25 fused to the C-terminal end of FtsZ | This study |
| pUT18FtsA | pUT18 containing the T18 fused to the C-terminal end of FtsA | This study |
| pUT18CFtsA | pUT18C containing the T18 fused to the N-terminal end of FtsA | This study |
| pKT25FtsA | pKT25 containing the T25 fused to the N-terminal end of FtsA | This study |
| pKNT25FtsA | pKNT25 containing the T25 fused to the C-terminal end of FtsA | This study |
| pUT18FtsE | pUT18 containing the T18 fused to the C-terminal end of FtsE | This study |
| pKNT25FtsE | pKNT25 containing the T25 fused to the C-terminal end of FtsE | This study |
| pUT18FtsQ | pUT18 containing the T18 fused to the C-terminal end of FtsQ | This study |
| pKNT25FtsQ | pKNT25 containing the T25 fused to the C-terminal end of FtsQ | This study |
| pUT18FtsI | pUT18 containing the T18 fused to the C-terminal end of FtsI | This study |
| pUT18CFtsI | pUT18C containing the T18 fused to the N-terminal end of FtsI | This study |
| pKT25FtsI | pKT25 containing the T25 fused to the N-terminal end of FtsI | This study |
| pKNT25FtsI | pKNT25 containing the T25 fused to the C-terminal end of FtsI | This study |
| pUT18FtsN | pUT18 containing the T18 fused to the C-terminal end of FtsN | This study |
| pUT18CFtsN | pUT18C containing the T18 fused to the N-terminal end of FtsN | This study |
| pKT25FtsN | pKT25 containing the T25 fused to the N-terminal end of FtsN | This study |
| pKNT25FtsN | pKNT25 containing the T25 fused to the C-terminal end of FtsN | This study |

| | | |
|---|---|---|
| pUT18ZipA | pUT18 containing the T18 fused to the C-terminal end of ZipA | This study |
| pUT18CZipA | pUT18C containing the T18 fused to the N-terminal end of ZipA | This study |
| pKT25ZipA | pKT25 containing the T25 fused to the N-terminal end of ZipA | This study |
| pKNT25ZipA | pKNT25 containing the T25 fused to the C-terminal end of ZipA | This study |
| pUT18SulA | pUT18 containing the T18 fused to the C-terminal end of SulA | This study |
| pKNT25SulA | pKNT25 containing the T25 fused to the C-terminal end of SulA | This study |
| pUT18ZapA | pUT18 containing the T18 fused to the C-terminal end of ZapA | This study |
| pUT18CZapA | pUT18C containing the T18 fused to the N-terminal end of ZapA | This study |
| pKT25ZapA | pKT25 containing the T25 fused to the N-terminal end of ZapA | This study |
| pKNT25ZapA | pKNT25 containing the T25 fused to the C-terminal end of ZapA | This study |

**Table S3:** List of plasmids used in this work.

**TableS4**

| Strain | Genotype | Source |
|---|---|---|
| *Escherichia coli* | | |
| DH5α | $F^-$, ϕ80d*lacZ*ΔM15 Δ(*lacZYA-argF*)*U169*, *endA1*, *recA1*, *hsdR17*($r_k^-m_k^+$), *supE44*, *thi-1*, Δ*gyrA96*, *relA1* | Laboratory stock |
| BL21(DE3) | $F^-$, *ompT*, *hsdS*$_B$($r_B^-m_B^-$), *gal*, *dcm* (DE3) | Laboratory stock |
| TOP10 | $F^{--}$, *mcr*A, Δ(*mrr-hsd*RMS-*mcr*BC), φ80*lac*ZΔM15, Δ*lac*X74, *rec*A1, *ara*D139, Δ(*ara-leu*)7697, *gal*U, *gal*K $\lambda^-$ *rps*L(Str$^r$), *end*A1, *nup*G | Laboratory stock |
| XL1Blue | *recA1*, *endA1*, *gyrA96*, *thi-1*, *hsdR17*, *supE44*, *relA1*, *lac* [*F´ proAB lacI*$^q$*Z*Δ*M15 Tn10* (Tet$^r$)]. | Laboratory stock |
| BTH101 | $F^-$, *cya-99*, *araD139*, *galE15*, *galK16*, *rpsL1* (Str$^r$), *hsdR2*, *mcrA1*, *mcrB1* | Euromedex |
| *Burkholderia cenocepacia* | | |
| J2315 | Wild type strain | Laboratory stock |
| K56-2 | Wild type strain | Laboratory stock |

**Table S4:** List of strains used in this work.

**Table S5**

| Primer | Sequence 5′- 3′ | Restriction site |
|---|---|---|

antibiotics MDPI

| | | |
|---|---|---|
| FtsZpUT18pKNT25For | GATTACGCCAAGCTTGATGGAATTCGAAATGCTGGA | *HindIII* |
| FtsZpUT18pKNT25Rev | GGTACCCGGGGATCCTCGTCAGCCTGCTTGCGCAGGAAAG | *BamHI* |
| FtsZpUT18CFor | GACTCTAGAGGATCCCATGGAATTCGAAATGCTGGA | *BamHI* |
| FtsZpUT18CRev | GAATTCGAGCTCGGTACCCGGTCAGCCTGCTTGCGCAGGAAAG | *KpnI* |
| FtsZpKT25For | GACTCTAGAGGATCCCATGGAATTCGAAATGCTGGA | *BamHI* |
| FtsZpkT25Rev | CTTAGTTACTTAGGTACCCGGTCAGCCTGCTTGCGCAGGA | *KpnI* |
| SulApUT18pKNT25For | GATTACGCCAAGCTTGATGCACCCCGCCCTCGCCCATCCTG | *HindIII* |
| SulApUT18Rev | CGGTACCCGGGGATCCTCGGCGACGGCGCCGGCGATCGTGGCC | *BamHI* |
| SulApKNT25Rev | GTACCCGGGGATCCTCGGCGACGGCGCCGGCGATCGTGGCC | *BamHI* |
| SulApUT18CpKT25For | GACTCTAGAGGATCCCATGCACCCCGCCCTCGCCCATCCT | *BamHI* |
| SulApUC18CRev | GTTATATCGATGAATTCGAGGCGACGGCGCCGGCGATCGTGGCC | *EcoRI* |
| SulApKT25Rev | GTTACTTAGGTACCCGGGCGACGGCGCCGGCGATCGTGGCC | *KpnI* |
| FtsApUT18pKNT25For | GATTACGCCAAGCTTGATGAGCAAAGACTACAAGGATC | *HindIII* |
| FtsApUT18pKNT2Rev | TTCGAGCTCGGTACCCGGAAGTTGCTCAGGAACC | *KpnI* |
| FtsApUT18CpKT25For | GACTCTAGAGGATCCCATGAGCAAAGACTACAAGGATC | *BamHI* |
| FtsApUT18CRev | GAATTCGAGCTCGGTACCCGGAAGTTGCTCAGGAACCATTCC | *KpnI* |
| FtsApKT25Rev | GTTACTTAGGTACCCGGAAGTTGCTCAGGAACCATTCC | *KpnI* |
| ZipApUT18pKNT25For | GATTACGCCAAGCTTGATGGACGAGTTGACACTCGGTTTG | *HindIII* |
| ZipApUT18pKNT25Rev | CGGGGATCCTCTAGAGTCTGGCTGAAGAGGCGGCGCGTGA | *XbaI* |
| ZipApUT18CpKT25For | GACTCTAGAGGATCCCATGGACGAGTTGACACTCGGTTTG | *BamHI* |
| ZipApUT18CRev | GAATTCGAGCTCGGTACCCGCTGGCTGAAGAGGCGGCGCGTGAC | *KpnI* |
| ZipApKT25Rev | GTTACTTAGGTACCCGCTGGCTGAAGAGGCGGCGCGTGAC | *KpnI* |
| ZapApUT18pKNT25For | GATTACGCCAAGCTTGATGAGCACCAAGCAGATCGAAGTCT | *HindIII* |
| ZapApUT18pKNT25rev | GTACCCGGGGATCCTCCTGCGTCTCGTGCTGTGCGAGC | *BamHI* |
| ZapApUT18CpKT25For | GACTCTAGAGGATCCCATGAGCACCAAGCAGATCGAAGTCT | *BamHI* |

| | | |
|---|---|---|
| ZapApUT18CRev | CGAGCTCGGTACCCGCTGCGTCTCGTGCTGTGCG AGC | *KpnI* |
| ZapApKT25Rev | GTTACTTAGGTACCCGCTGCGTCTCGTGCTGTGC GAGC | *KpnI* |
| FtsEpUT18pKNT25For | GATTACGCCAAGCTTGATGATCCGCCTCGAACGC ATCGAC | *HindIII* |
| FtsEpUT18pKNT25Rev | GTACCCGGGGATCCTCGAACGCCGGCACGCCTT GCGCGAG | *BamHI* |
| FtsEpUT18CpKT25For | GACTCTAGAGGATCCCATGATCCGCCTCGAACG CATCGAC | *BamHI* |
| FtsEpUT18CRev | CGAGCTCGGTACCCGGAACGCCGGCACGCCTTG CGCGAG | *KpnI* |
| FtsEpKT25Rev | GTTACTTAGGTACCCGGAACGCCGGCACGCCTTG CGCGAG | *KpnI* |
| FtsQpUT18pKNT25For | GATTACGCCAAGCTTGATGTGGAACAACGTTCG CCAAC | *HindIII* |
| FtsQpUT18pKNT25Rev | GTACCCGGGGATCCTCCTTCTTGCGCTTGTCGGT ATCG | *BamHI* |
| FtsQpUT18CpKT25For | GACTCTAGAGGATCCCATGTGGAACAACGTTCG CCAAC | *BamHI* |
| FtsQpUT18CRev | CGAGCTCGGTACCCGCTTCTTGCGCTTGTCGGTA TCG | *KpnI* |
| FtsQpKT25Rev | GTTACTTAGGTACCCGCTTCTTGCGCTTGTCGGTA TCG | *KpnI* |
| FtsIpUT18pKNT25for | GATTACGCCAAGCTTGATGAAGCCGTCCCAGAA GCGC | *HindIII* |
| FtsIpUT18pKNT25rev | GTACCCGGGGATCCTCTCGAACTACTCCTGGTGA ATTAC | *BamHI* |
| FtsIpUT18CFor | CAGGTCGACTCTAGAGATGAAGCCGTCCCAGAA GCGC | *XbaI* |
| FtsIpUT18CRev | GTTATATCGATGAATTCGATCGAACTACTCCTGG TGAATTAC | *EcoRI* |
| FtsIpKT25For | GGGTCGACTCTAGAGATGAAGCCGTCCCAGAAG CGC | *XbaI* |
| FtsIpKT25Rev | AACGACGGCCGAATTCTCATCGAACTACTCCTG GTGAATTAC | *EcoRI* |
| FtsNpUT18pKNT25For | GATTACGCCAAGCTTGGTGCTGGGCCTGATCGTC GGCCTCG | *HindIII* |
| FtsNpUT18pKNT25Rev | GTACCCGGGGATCCTCCTGCTTCGTGAAGCGGAT CACC | *BamHI* |
| FtsNpUT18CpKT25For | GACTCTAGAGGATCCCGTGCTGGGCCTGATCGTC GGCCTCG | *BamHI* |
| FtsNpUT18CRev | CGAGCTCGGTACCCGCTGCTTCGTGAAGCGGATC ACC | *KpnI* |
| FtsNpKT25Rev | GTTACTTAGGTACCCGCTGCTTCGTGAAGCGGAT CACC | *KpnI* |
| pUT18pKNT25CheckFor | CTTTATGCTTCCGGCTCG | |
| pUT18CheckRev | GTTCGCGATCCAGGCCGC | |
| pKNT25CheckRev | GCGTAACCAGCCTGATGCG | |

| | | |
|---|---|---|
| pUT18CCheckFor | GTCACCCGGATTGCGGCG | |
| pUT18CCheckRev | GTGTCGGGGCTGGCTTAAC | |
| pKT25CheckFor | GCAGTTCGGTGACCAGCGG | |
| pKT25CheckRev | GCAAGGCGATTAAGTTGGG | |

**Table S5:** List of primers used for cloning the divisome genes into the BACTH plasmids. The restriction site is underlined.
